# Benchmarking deep learning-based reconstruction methods for photoacoustic computed tomography with clinically relevant synthetic datasets

**DOI:** 10.1117/1.JBO.32.S1.S14903

**Published:** 2026-10-14

**Authors:** Panpan Chen, Seonyeong Park, Gangwon Jeong, Refik Mert Cam, Umberto Villa, Mark A. Anastasio

**Affiliations:** a University of Illinois Urbana-Champaign, Department of Bioengineering, Urbana, Illinois, United States; b Washington University in St. Louis, Mallinckrodt Institute of Radiology, St. Louis, Missouri, United States; c University of Illinois Urbana-Champaign, Department of Electrical and Computer Engineering, Urbana, Illinois, United States; d The University of Texas at Austin, Department of Biomedical Engineering, Austin, Texas, United States

**Keywords:** photoacoustic computed tomography, optoacoustic computed tomography, benchmarking, deep learning, image reconstruction, virtual imaging studies

## Abstract

**Significance:**

Deep learning (DL)-based image reconstruction methods for photoacoustic computed tomography (PACT) have developed rapidly in recent years. However, most existing methods have not employed standardized datasets, and their evaluations rely on traditional image quality (IQ) metrics that may lack clinical relevance. The absence of a standardized framework for clinically meaningful IQ assessment hinders fair comparison and raises concerns about the reproducibility and reliability of reported advancements in PACT.

**Aim:**

A benchmarking framework is proposed that provides open-source, anatomically plausible synthetic datasets and evaluation strategies for DL-based acoustic inversion methods in PACT.

**Approach:**

The datasets each include over 10,000 two-dimensional (2D) stochastic breast objects with clinically relevant lesions and paired measurements at varying modeling complexity. The evaluation strategies incorporate both traditional and task-based IQ measures to assess fidelity and clinical utility. A preliminary benchmarking study is conducted to demonstrate the framework’s utility by comparing DL-based and physics-based reconstruction methods.

**Results:**

The benchmarking study demonstrated that the proposed framework enabled comprehensive, quantitative comparisons of reconstruction performance and revealed important limitations in certain DL-based methods. Although they performed well according to traditional IQ measures, they often failed to accurately recover lesions. This highlights the inadequacy of traditional metrics and motivates the need for task-based assessments.

**Conclusions:**

The proposed benchmarking framework enables systematic comparisons of DL-based acoustic inversion methods for 2D PACT. By integrating clinically relevant synthetic datasets with rigorous evaluation protocols, it enables reproducible, objective assessments and facilitates method development and system optimization in PACT.

## 1 Introduction

Photoacoustic computed tomography (PACT), also known as optoacoustic tomography, is an emerging imaging modality that exploits optical contrast mechanisms and ultrasound detection principles.^1^ In PACT, biological tissues are irradiated with a short laser pulse, resulting in the absorption of optical energy within the tissues. Under thermal confinement conditions, the absorbed optical energy induces the generation of acoustic pressure wavefields via the photoacoustic effect.^2^ These pressure waves propagate outward and are subsequently detected by broadband ultrasonic transducers positioned along the measurement surface. From these measurements, tomographic reconstruction methods can be employed to estimate the spatially varying initial pressure distribution,^3^ which is proportional to the deposited optical energy density. The hybrid nature of PACT, which combines optical contrast with ultrasonic detection, provides excellent endogenous optical absorption contrast at high resolution, establishing it as a promising technology for diverse anatomical, functional, and molecular imaging applications in both preclinical research and clinical medicine.^4^

Various physics-based reconstruction methods have been proposed for PACT^5^^–^^9^. However, many of these approaches face significant challenges in practical imaging scenarios. One challenge involves reconstructing images from incomplete measurements, typically referred to as limited-view and sparse-view problems.^10^^,^^11^ Such conditions lead to inaccurate reconstructions with artifacts. Limited-view problems arise when the angular coverage of the detection aperture is restricted, whereas sparse-view problems occur when spatial sampling is insufficient. Furthermore, conventional reconstruction algorithms often assume point-like transducers, neglecting finite transducer aperture sizes and constrained scanning geometries. This introduces spatially variant system responses that deviate from the assumed linear shift-invariant model, causing resolution degradation and geometric distortions.^12^ Moreover, acoustic heterogeneity presents another challenge. Because biological tissues exhibit spatially varying acoustic properties with frequency-dependent attenuation, methods based on the assumption of an acoustically homogeneous, lossless medium fail to account for these variations and consequently can produce distortions that become severe when acoustic heterogeneity is pronounced.^3^ Although recent model-based methods incorporating more accurate physical modeling have been proposed to compensate for these limitations, such approaches are generally computationally intensive.^9^^,^^13^^,^^14^

Recently, in addition to the well-established physics-based approaches, deep learning (DL)-based image reconstruction methods have been actively developed for PACT.^15^^,^^16^ As data-driven approaches, DL methods can leverage learned data-driven priors to model complex mappings to enhance image reconstruction quality under non-ideal conditions and enable near real-time imaging through rapid inference.^17^^–^^20^ These methods can be broadly categorized into supervised, semi-supervised, and unsupervised approaches, which differ primarily in their dependence on labeled data.^16^ The performance of these paradigms depends critically on large, diverse, and representative training datasets that capture application-specific variability to characterize the statistical distribution of the target domain.^15^ For instance, in computer vision, benchmark datasets such as ImageNet^21^ have driven rapid progress by providing large-scale, publicly available data for model training and performance comparison. However, only a few open-source datasets applicable to PACT are currently available.^22^^–^^24^

To effectively support the development of artificial intelligence in PACT, a dataset comprised of well-characterized (to-be-imaged) objects and their corresponding tomographic data should meet the following requirements: (1) objects should exhibit realistic anatomical variability; (2) objects should represent plausible physiological, acoustic, and optical property variations; (3) objects should incorporate clinically relevant pathological features, such as lesions, to enable task-based image quality (IQ) assessments; and (4) the measurement data shall be acquired using a specified imaging system configuration and must embody the underlying imaging physics, including inherent uncertainties and errors.

Experimental datasets of raw PACT measurements have been considered for benchmarking purposes.^22^^,^^25^ For example, Ozdemir et al.^24^ recently introduced a large-scale forearm dataset comprised of more than 70,000 *in vivo* photoacoustic measurements acquired under various imaging configurations. However, it is often infeasible to obtain ground truth from *in vivo* experimental datasets, which is essential for supervised learning.^26^ Moreover, the collection of large-scale clinical measurement data is difficult, particularly for emerging modalities such as PACT. Although well-characterized physical phantoms can provide ground truth data, the construction and imaging of a large and diverse ensemble of experimental phantoms is time-consuming and costly.^27^^,^^28^

In contrast, synthetic PACT datasets comprised of paired objects and corresponding simulated measurements offer a flexible and cost-effective alternative.^29^^,^^30^ Through stochastic generation of numerical phantoms that are virtually imaged using high-fidelity multiphysics modeling of the PACT forward process, large-scale ensembles of objects and corresponding measurement data can be generated readily with known ground truth by design. Such approaches afford complete control over anatomical, physiological, and pathological variability while maintaining realistic tissue property distributions and incorporating disease-specific features.^31^ The resulting well-characterized datasets serve as a solid basis for comprehensive benchmarking.

This study proposes a benchmarking framework for PACT image reconstruction that integrates open-source synthetic data and objective IQ assessment methodologies based on lesion detection tasks. Motivated by a 2D ring-array imaging system,^32^ two-dimensional (2D) PACT is considered in this work. However, the framework may also find application for early-stage prototyping of methods that can eventually be extended to use with 3D PACT systems.^16^^,^^33^^,^^34^ The synthetic datasets comprise realistic 2D breast object representations derived from previously developed three-dimensional (3D) numerical breast phantoms (NBPs),^31^ which reflect clinically relevant anatomical variations and photoacoustic tissue properties. A high-fidelity virtual imaging framework is implemented to simulate photoacoustic measurements. The evaluation strategies integrate both traditional and task-based measures of IQ, facilitating the assessment of physical and statistical properties of reconstructed images along with their utility for diagnostic tasks. The proposed datasets and code packages for the evaluation strategies are publicly available.^35^^,^^36^

The remainder of the paper is organized as follows. In Sec. 2, the PACT imaging model, the motivation for virtual imaging studies, and the salient aspects of task-based IQ assessment are reviewed. The proposed benchmarking framework is detailed in Sec. 3. Example benchmarking studies that demonstrate the utility of the proposed benchmarking framework are described in Sec. 4, with the corresponding results are provided in Sec. 5. Finally, the paper concludes with a discussion in Sec. 6.

## 2 Background

This section reviews the PACT imaging model, virtual imaging studies for PACT, and task-based IQ assessment methods.

### 2.1 Photoacoustic Computed Tomography

In PACT, the sought-after object is irradiated by a short laser pulse, resulting in optical energy absorption denoted by A(r), where $r\in R^{3}$ represents the spatial position. Through the photoacoustic effect, this absorbed energy induces a localized acoustic pressure rise, generating an initial pressure distribution $p_{0}(r)=\Gamma (r)A(r)$, where Γ(r) is the dimensionless Grüneisen parameter. The resulting initial pressure distribution subsequently propagates as acoustic pressure waves p(r,t) through the object and surrounding medium. These waves are recorded by ultrasonic transducers positioned on a measurement aperture, yielding the pressure measured at the transducer locations. The detected pressure is recorded over the time interval t∈[0,T], where t=0 denotes the laser excitation time and T is the end of the acquisition period.

For lossy, acoustically heterogeneous media, the propagation of p(r,t) can be described by the following coupled system of first-order partial differential equations:^37^

$$\frac{\partial }{\partial t}u(r,t)=-\frac{1}{\rho_{0}(r)}\nabla p(r,t),\;\frac{\partial }{\partial t}\rho (r,t)=-\rho_{0}(r)\nabla \cdot u(r,t),\;p(r,t)=c_{0}(r{)}^{2}\{1-\mu (r)\frac{\partial }{\partial t}(-\nabla^{2}{)}^{y/2-1}-\eta (r)(-\nabla^{2}{)}^{(y-1)/2}\}\rho (r,t),$$ (1)

subject to the initial conditions:

$${p_{0}(r)\equiv p(r,t)|}_{t=0}=\Gamma (r)A(r),\;{u(r,t)|}_{t=0}=0.$$ (2)

Here, u(r,t) represents the acoustic particle velocity, ρ(r,t) is the acoustic density distribution, $c_{0}(r)$ denotes the speed-of-sound (SOS) distribution, and $\rho_{0}(r)$ is the ambient density distribution. The parameter y is the power-law exponent characterizing the frequency dependence of acoustic attenuation, such that $\alpha (r,f)=\alpha_{0}(r)|f{|}^{y}$, where $\alpha_{0}(r)$ is the frequency-independent attenuation coefficient, and f denotes the temporal frequency. In this study, y was assumed to be spatially homogeneous within each breast phantom and was assigned according to the breast type, with values of 1.1151, 1.1642, 1.2563, and 1.3635 for breast types A, B, C, and D, respectively.^31^ The quantities μ(r) and η(r) describe the acoustic absorption and dispersion proportionality coefficients:

$$\mu (r)=-2\alpha_{0}(r)c_{0}(r{)}^{y-1},\;\eta (r)=2\alpha_{0}(r)c_{0}(r{)}^{y}\;\tan(\frac{\pi y}{2}).$$ (3)

From the measured acoustic data, the initial pressure distribution $p_{0}(r)$ can be reconstructed by solving an acoustic inverse problem.^3^ Numerous reconstruction methods have been developed for PACT, which can be broadly categorized into physics-based and data-driven approaches.^3^^,^^16^ Physics-based methods include analytical methods, non-iterative numerical methods (such as time-reversal algorithms), and iterative optimization-based methods,^5^^,^^8^^,^^38^ all of which leverage established imaging physics to achieve robust reconstruction. However, analytical methods often exhibit degraded performance in nonideal scenarios, whereas iterative methods require high computational cost. Alternatively, DL-based reconstruction methods adopt a data-driven paradigm to learn the inverse mappings without explicit physical modeling.^20^^,^^33^^,^^39^^,^^40^ Although such DL-based methods demonstrate potential for achieving both computational efficiency and reconstruction accuracy in complex scenarios, their stability and generalizability remain open challenges.

### 2.2 Virtual Imaging Studies for PACT

Systematic and rigorous evaluation of PACT reconstruction methods is essential for their development and optimization toward clinical translation. However, directly assessing algorithm performance in experimental studies is challenging, as the experimental data generally do not provide ideal ground truth for quantitative evaluation of reconstruction performance. Particularly, *in vivo* studies pose challenges in determining ground truth and are constrained by ethical considerations. Although physical phantoms can offer known ground truth, their construction is often costly, and factors such as system calibration errors and measurement noise can introduce uncertainties that limit their reliability. In contrast, virtual imaging (VI) studies provide a cost-effective and controlled alternative for systematic evaluation of PACT reconstruction methods. By employing synthetic objects and simulated measurement data, VI studies allow precise control of parameters to assess and refine methods. In addition, because the ground truth is explicitly defined, VI studies support quantitative and objective assessments of IQ that align algorithm development with clinical requirements.

A VI study pipeline for PACT integrates numerical phantoms, PACT forward process modeling for the target imaging system, and quantitative IQ evaluation. Numerical phantoms, constructed through mathematical or computational modeling of anatomical structures with tissue-specific physiological and photoacoustic properties, provide the ground truth. To ensure clinical relevance, numerical phantoms must realistically capture stochastic anatomical and physiological variations, including both intrinsic tissue characteristics and pathology-associated alterations.^30^^,^^31^ In PACT, several NBPs have been developed,^29^^,^^31^^,^^41^^,^^42^ which correspond to representative examples of such numerical phantoms.

Virtual PACT imaging involves a two-stage physics-based modeling framework that incorporates both optical and acoustic processes.^2^ In the optical stage, photon transport through biological tissues is simulated to compute the spatial distribution of absorbed optical energy A(r). Methods such as Monte Carlo techniques (e.g., MCX^43^) or diffusion approximations^44^ account for wavelength-dependent optical properties and realistic illumination conditions to generate the initial pressure distribution $p_{0}(r)$. In the acoustic stage, acoustic wave propagation is simulated to obtain the pressure measurements acquired by transducers p(r,t) according to Eqs. (1) and (2), while considering acoustic medium properties, detection geometry, and transducer response. Established tools such as k-Wave^45^ can be used for customizable acoustic simulations that closely replicate experimental conditions.

### 2.3 Task-Based Image Quality Assessment

Quantitative evaluation plays a central role in evaluating and optimizing reconstruction algorithms for PACT. Physical measures of IQ have been widely employed in PACT imaging, including spatial resolution, contrast-to-noise ratio (CNR),^46^^,^^47^ structural similarity index (SSIM),^48^^,^^49^ and mean squared error (MSE).^48^ These traditional metrics provide standardized benchmarks for assessing image fidelity, noise characteristics, and structural preservation in reconstructed images. However, although these physical IQ measures effectively quantify certain image properties, they do not always correlate with the utility of images with consideration of a specified diagnostic task such as lesion detection. This limitation motivates the adoption of task-based measures of IQ, which are introduced in the following section.

Task-based IQ assessment provides a rigorous framework for developing and optimizing PACT reconstruction methods by directly quantifying image utility for specific clinical objectives.^50^^,^^51^ In contrast to traditional IQ metrics, task-based measures of IQ quantify the utility of an image for a specified clinical purpose based on observer performance..^52^^,^^53^ Here, the observer refers to the entity that performs the clinically relevant task. Although the observer can be a human reader, early-stage assessments via VI studies typically employ numerical observer models. These are computer algorithms to enable objective, reproducible, and computationally efficient evaluations.^54^^,^^55^ Clinical tasks can be broadly categorized into three types: estimation tasks, where the objective is to measure a parameter of interest; classification tasks, where the goal is to discriminate among multiple possible states (e.g., presence or absence of a lesion); and hybrid tasks that combine multiple objectives (e.g., detection-estimation). This work focuses exclusively on classification tasks.

In classification tasks, the observer must make a decision among multiple hypotheses $\left\{H_{0},H_{1},\ldots ,H_{n_{C}-1}\right\}$, where each hypothesis $H_{i}$ represents a distinct clinical condition and $n_{C}$ denotes the total number of classes. For example, in a binary lesion detection task, the observer chooses between signal-present ($H_{1}$) and signal-absent ($H_{0}$) hypotheses. The observer makes decisions by first computing a test statistic $t_{i}(g)$ that maps the observed data vector g (i.e., reconstructed images or measurement data) to a real-valued scalar variable. The observer then applies a decision rule to select the hypothesis with the maximum test statistic:^56^

$$\text{Decide }H_{i}\text{ if }t_{i}(g)>t_{j}(g),\;\forall \;j\neq i.$$ (4)

In the special case of binary classification (i.e., $n_{C}=2$), the decision rule simplifies to a comparison between a single test statistic t=t(g) and a decision threshold τ:

$$\mathrm{Decide}\;H_{1}\text{ if }t(g)>\tau ;\;\text{otherwise decide }H_{0}.$$ (5)

Various numerical observers have been developed for objective IQ assessment.^52^^,^^57^ The ideal observer^54^ is a specific type of numerical observer that implements an optimal decision strategy. However, this approach requires complete knowledge of image statistics and is generally difficult to compute. Linear observers offer practical alternatives, with the Hotelling observer (HO) serving as the optimal linear discriminant based on first- and second-order statistical moments.^58^ As an extension to HO, the channelized Hotelling observer (CHO) incorporates channels to reduce data dimensionality while preserving task-relevant information. Depending on the task, different figures of merit (FOMs) can be employed to evaluate the observer performance.^59^ For binary detection tasks, commonly used measures of signal detection or discrimination performance include the signal-to-noise ratio of the test statistic (${\mathrm{SNR}}_{t}$) and the area under the receiver operating characteristic curve (AUC).

## 3 Proposed Benchmarking Framework

This section introduces the proposed benchmarking pipeline for assessing PACT image reconstruction methods, comprising clinically relevant synthetic breast datasets^60^ and evaluation strategies that integrate both physical and task-based measures of IQ.

### 3.1 Proposed Synthetic Datasets for Breast PACT

As shown in Table 1, three synthetic datasets for 2D breast PACT were generated with progressively increasing acoustic modeling complexity. Each dataset consists of 2D stochastic breast objects and corresponding measurements. Dataset 1 assumes a homogeneous acoustic medium with point-like transducers; Dataset 2 incorporates acoustic heterogeneity while retaining point-like transducers; and Dataset 3 employs both acoustic heterogeneity and finite-sized transducers. For data generation, previously developed stochastic 3D NBP data^31^ were utilized, which included corresponding initial pressure distributions obtained from 3D photon transport simulations.^61^ In this study, 2D objects were derived from the 3D NBP data, and subsequently, the acoustic measurement acquisition process was modeled in 2D.

**Table 1:** Summary of the generated breast datasets for PACT.

Dataset	Size	Acoustic heterogeneity	SIR^a^	Object			Measurement
				Composition	BI-RADS density distribution	Lesion distribution	
Dataset 1		✗	✗				
Dataset 2	10,904	✓	✗	Initial pressure map Speed-of-sound map Acoustic density map Acoustic attenuation coefficient map	Type A: 1240 Type B: 3516 Type C: 4324 Type D: 1824	Present: 2726 Paired absent: 2726 Unpaired absent: 5452	Sampling rate: 40 MHz Geometry: ring array Densely sampled, noiseless data
Dataset 3		✓	✓				

a SIR: transducer spatial impulse response.

Each dataset comprises 10,904 samples, with each sample consisting of an object and its corresponding measurement data. Specifically, the dataset includes 2726 lesion-present samples, 2726 paired lesion-absent samples that serve as matched controls for the lesion-present samples in task-based IQ assessment, and 5452 unpaired lesion-absent samples that provide additional anatomical diversity for training DL models. To reflect clinical breast density variations, the objects span four Breast Imaging Reporting and Data System (BI-RADS) categories:^62^ (A) almost entirely fatty, (B) scattered fibroglandular density, (C) heterogeneously dense, and (D) extremely dense breast tissue. A summary of the dataset composition is provided in Table 1.

The entire dataset generation pipeline consists of three main stages:^60^ 1. generation of 3D NBPs and simulation of their initial pressure distributions, 2. generation of 2D objects, and 3. simulation of acoustic measurements using a 2D forward model.

#### 3.1.1 3D numerical breast phantoms generation and initial pressure simulation

The stochastic 3D NBPs developed by Park et al.^31^ formed the basis of the proposed datasets, which were designed to faithfully represent various breast types through their clinically relevant variability in anatomical structures and functional, optical, and acoustic properties. In this study, 1500 pairs of stochastic 3D healthy and lesion-inserted NBPs were used to generate the objects in the proposed dataset. Figures 1 and 2(a) present the 3D tissue label map of an example NBP, illustrating its anatomical structure. Each NBP corresponds to one of the four BI-RADS density categories, with statistically defined breast size and shape parameters, physiologically realistic blood vasculature modeling, and diverse tissue compositions including skin, muscle, blood vessels, and fat. For lesion-present NBPs, a numerical lesion phantom (NLP) was constructed, which contains a viable tumor cell region characterized by irregular, spiculated boundaries, as well as a necrotic core and a peripheral angiogenesis region.

**Fig. 1:**
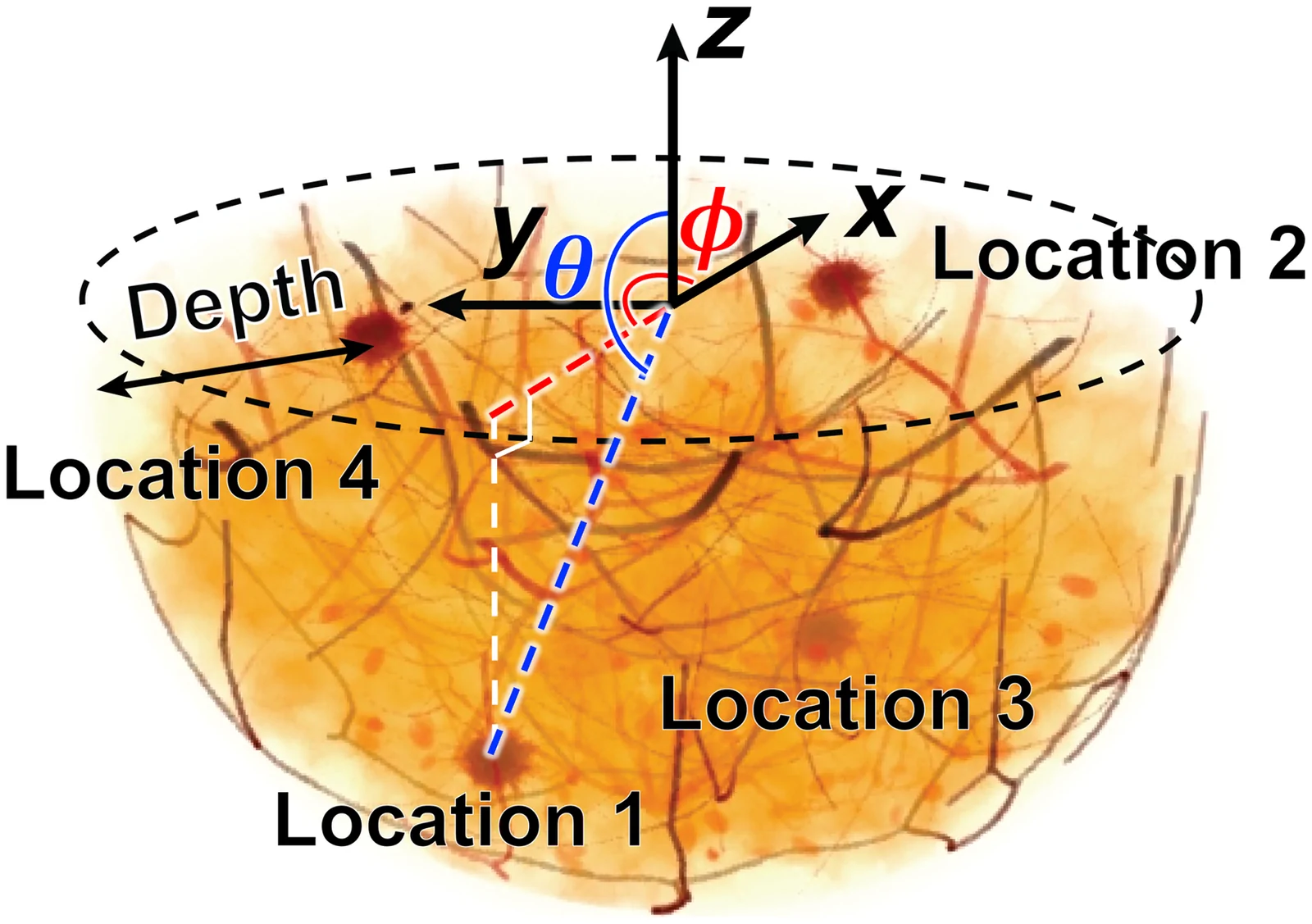
3D breast tissue label map with a numerical lesion model inserted in four predetermined locations. Lesions at Locations 1 and 2 are positioned at a depth of 10 mm, and those at Locations 3 and 4 at 20 mm, representing shallow and deep cases for assessing light fluence attenuation with depth. Polar angles (θ) of 100 deg and 140 deg correspond to regions near the chest wall and nipple, respectively, enabling evaluation under distinct illumination conditions, while azimuthal angles (ϕ) are separated to minimize optical interference within the same NBP.

**Fig. 2:**
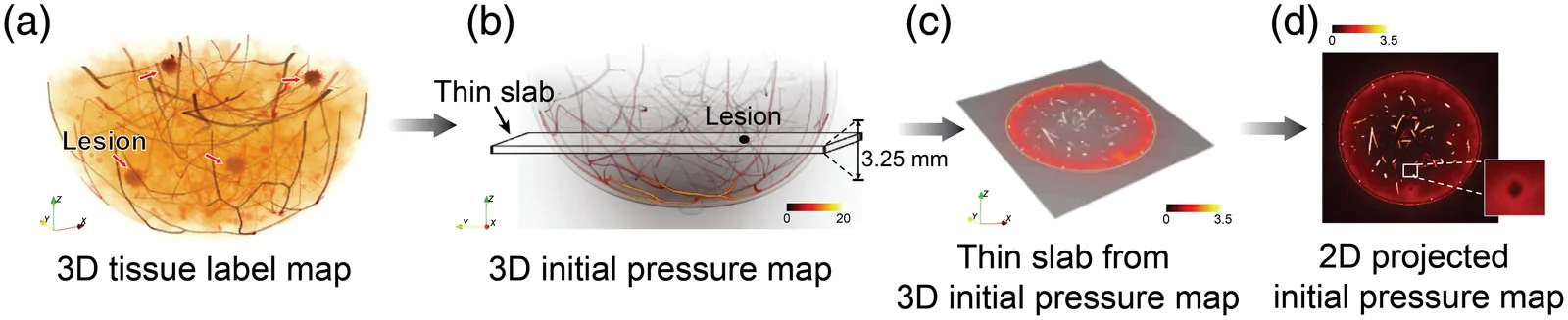
2D object generation process. (a) A 3D tissue label map of a stochastic NBP is used to assign optical and acoustic properties. (b) A 3D initial pressure map is generated via photon transport simulation based on the optical property distributions. (c) A thin slab is extracted from the 3D initial pressure map. (d) The slab is averaged along the z-axis to produce a 2D initial pressure map.

As illustrated in Fig. 1, four candidate locations were predefined within each NBP in spherical coordinates relative to the phantom center: Location 1 (10 mm, 140 deg, 180 deg), Location 2 (10 mm, 100 deg, 0 deg), Location 3 (20 mm, 140 deg, 270 deg), and Location 4 (20 mm, 100 deg, 90 deg), where the coordinates represent depth, polar angle θ, and azimuthal angle ϕ, respectively. For each NBP, the lesion was randomly inserted at one to four of the predefined candidate locations. These locations were selected to systematically investigate depth-dependent optical fluence attenuation at 10 and 20 mm, with polar angles chosen to capture distinct illumination conditions: lesions positioned at 100 deg near the chest wall experience reduced fluence due to limited light penetration, whereas those at 140 deg closer to the nipple region receive enhanced illumination. The azimuthal angles were selected to ensure that the optical fluence distribution around each lesion would remain independent and minimally affected by neighboring lesions within the same NBP.

Through stochastic property assignment, 3D distributions of optical and acoustic properties were obtained. To simulate initial pressure distributions, photon transport simulations were performed in 3D using MCX software,^43^ based on the 3D optical property distributions and an assumed configuration of the light delivery system. The illumination geometry included 20 evenly positioned arc-shaped illuminators (radius of 145 mm, central angle of 80 deg), each equipped with five linear fiber-optic segments along its surface. Each segment was represented by a line beam with a conical angular distribution characterized by a half-angle of 12.5. Additional details on property assignment and optical simulation can be found in References 31, 61.

#### 3.1.2 Generation of 2D objects

As shown in Figs. 2(b)–2(d), 2D objects were generated from the 3D initial pressure maps and corresponding 3D acoustic property maps to construct the proposed datasets. To approximately emulate the effect of the elevational focusing effect of transducers in 2D PACT systems, 2D initial pressure distributions were derived by averaging thin slabs (3.25 mm thickness) from the 3D initial pressure maps along the z-axis, rather than extracting single slices. Three types of 2D initial pressure distributions were generated based on the z-position of the selected 3D slabs relative to the predetermined candidate lesion locations. Slabs centered at these candidate z-positions were used to produce *paired* datasets, consisting of *paired lesion-present* and *lesion-absent* slices derived from corresponding 3D lesion-present and healthy phantoms, respectively. These matched pairs enabled direct comparison in signal detection analysis. In contrast, slabs extracted from 3D healthy phantoms and centered at z-positions offset by ±5  mm relative to the candidate lesion locations were used to generate *unpaired lesion-absent* slices that introduced additional anatomical variability into the datasets. Similarly, 2D acoustic property maps were obtained by averaging thin slabs from the corresponding 3D acoustic property maps along the z-axis. This approach was motivated by prior work,^63^ which demonstrated that effective 2D acoustic property distributions can be approximated as weighted averages of the underlying 3D distributions. Each resulting object was characterized by 2D maps of initial pressure, SOS, density, and acoustic attenuation, all with a resolution of 1360×1360  pixels and a pixel size of 0.125 mm. Figure 3 shows the representative 2D lesion-present objects corresponding to the four breast density types.

**Fig. 3:**
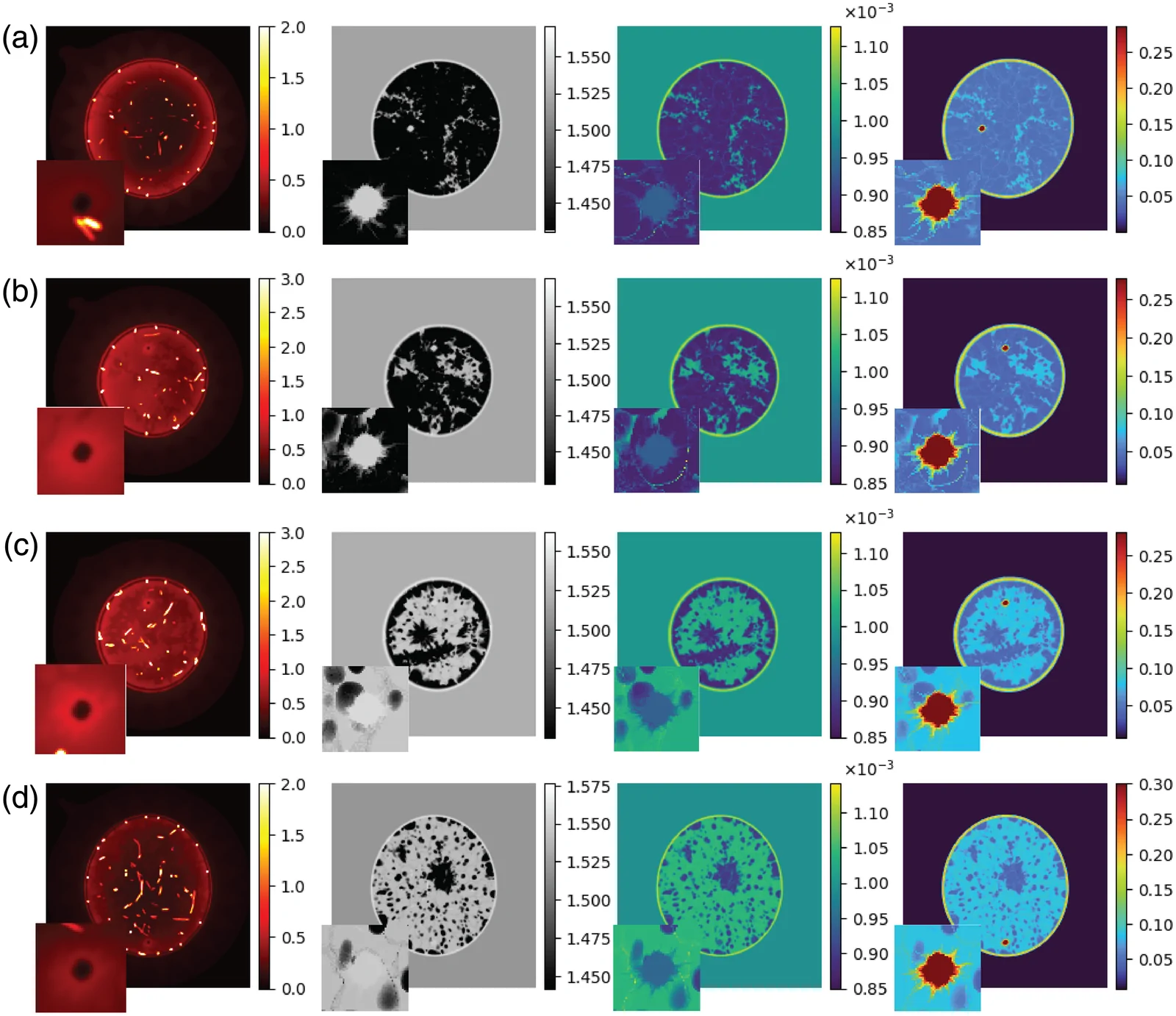
Representative 2D lesion-present object examples from four breast density types. From left to right: initial pressure distribution, SOS, acoustic density map, and attenuation coefficient map. Lesion region is zoomed in. From up to bottom: (a) almost entirely fatty, (b) scattered areas of fibroglandular density, (c) heterogeneously dense, and (d) extremely dense.

#### 3.1.3 Acoustic measurements simulation using 2D forward model

As shown in Fig. 4, PACT measurement data were acquired for each object via 2D acoustic wave propagation simulation using the k-Wave toolbox,^45^ assuming a circular measurement geometry with a radius of 85 mm. The sampling rate was set to 40 MHz with a computational grid size of 0.125 mm. Dataset 1 represents a highly stylized scenario with 1800 point-like transducers uniformly distributed along the measurement circle and an acoustically homogeneous lossless medium, characterized by a uniform SOS of 1.5  mm/μs and a density of $1\times 10^{-3}\;g/{\mathrm{mm}}^{3}$. Dataset 2 introduces greater realism by incorporating spatially varying SOS, density, and acoustic attenuation properties from the extracted 2D acoustic property maps while retaining the 1800 point-like transducer configuration. Dataset 3 employs the most realistic model, including both acoustic heterogeneity and the SIR of the finite-sized transducers (6 mm in length) arranged in a 360-element ring array.

**Fig. 4:**
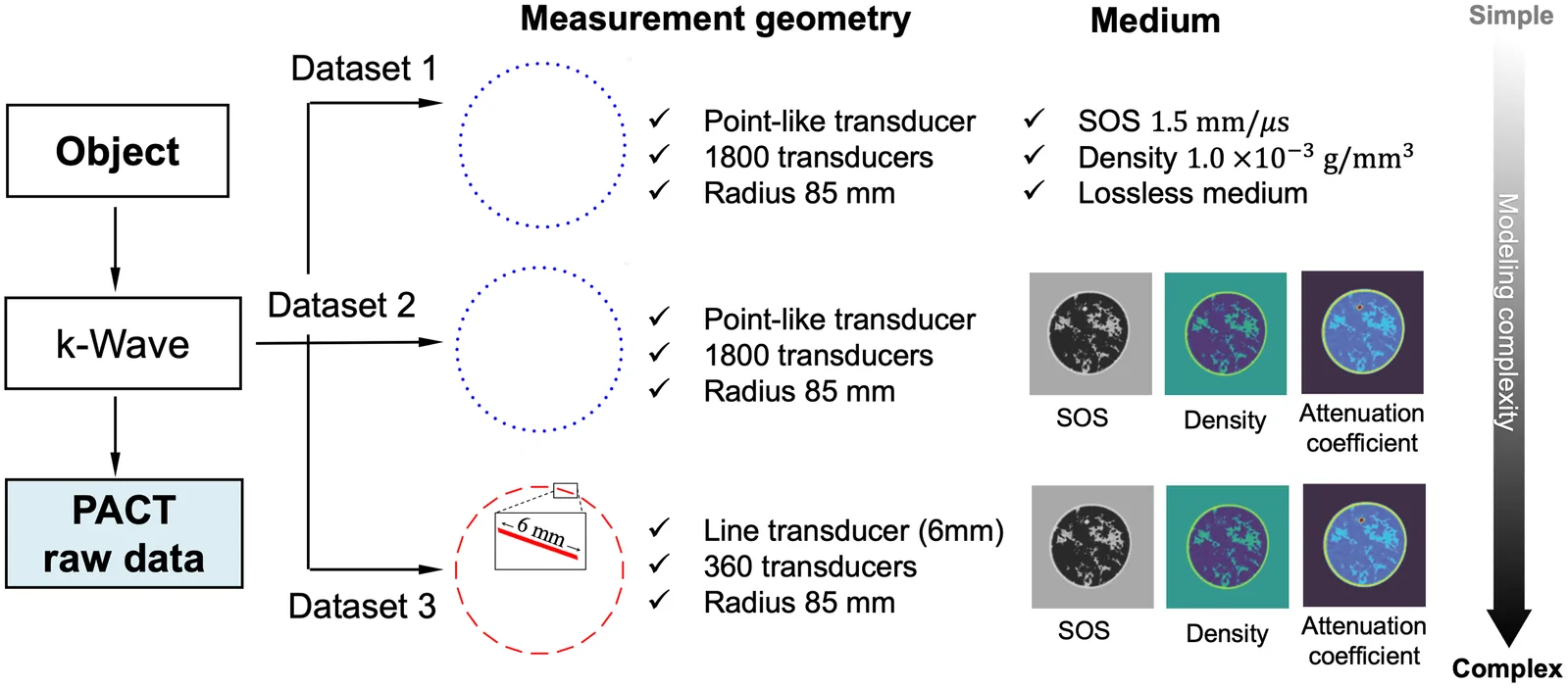
Virtual PACT measurement acquisition designs for the three datasets. Three datasets represent increasing modeling complexity, from an acoustically homogeneous medium with point-like transducers to an acoustically heterogeneous medium with finite-size line transducers.

#### 3.1.4 Statistical properties of generated breast datasets

The datasets incorporate stochastic variations in anatomical structures and acoustic parameters to represent physiological variations in breast tissue. Table 2 presents the statistical distributions of tissue parameters across all generated objects. Here, S denotes the breast cross-sectional area, and $f_{fg}$ represents the breast density percentage (i.e., the ratio of fibroglandular tissue to total breast area). This variability provides a comprehensive representation of breast tissue characteristics to support the training of DL models as well as the development and evaluation of image reconstruction methods.

**Table 2:** Statistical distribution of tissue parameters in the generated breast PACT datasets.

	Anatomical information		Acoustical information		
	S [${\mathrm{mm}}^{2}$]	$f_{fg}$ [%]	$c_{0}$ [mm/μs]	$\rho_{0}$ [$g/{\mathrm{mm}}^{3}$]	$\alpha_{0}$ [$\mathrm{dB}/{\mathrm{MHz}}^{y}\cdot \mathrm{mm}$]
Mean	9298.67	58.30	1.48	$9.66\times 10^{-4}$	$6.09\times 10^{-2}$
Std	2699.79	19.60	0.03	$4.43\times 10^{-5}$	$1.03\times 10^{-2}$
Min	3380.97	17.40	1.43	$8.47\times 10^{-4}$	$3.60\times 10^{-2}$
Max	18,596.97	89.10	1.55	$1.07\times 10^{-3}$	$9.77\times 10^{-2}$

### 3.2 Proposed Evaluation Strategies

The proposed evaluation strategies include both traditional and task-based IQ assessment metrics to enable clinically meaningful evaluations of reconstruction performance.

#### 3.2.1 Traditional measures of image quality

Traditional measures of IQ provide full-reference quantitative assessments of reconstruction fidelity. The study incorporates two standard metrics: relative MSE and SSIM.^64^ The relative MSE was computed as the MSE divided by the mean value of the true object, accounting for the stochastic variability in breast types and tissue properties within the datasets, and is given by

$$\text{Relative MSE}=\frac{\|f^{\text{true}}-f{\|}_{2}^{2}}{\|f^{\text{true}}{\|}_{2}^{2}}=\frac{\overset{N}{\underset{i=1}{\sum }}(f_{i}^{\text{true}}-f_{i}{)}^{2}}{\overset{N}{\underset{i=1}{\sum }}(f_{i}^{\text{true}}{)}^{2}},$$ (6)

where $f^{\text{true}}\in R^{N}$ denotes the true object and $f\in R^{N}$ represents the reconstructed image, with N denoting the number of pixels in the discretized image representation. For simplicity, relative MSE is referred to as MSE throughout this study.

#### 3.2.2 Task-based measures of image quality

To assess the diagnostic relevance of the reconstructed images, each reconstruction method was evaluated on two diagnostic tasks using datasets containing objects with lesions positioned at one of four predefined lesion sites (as detailed in Sec. 3.1.1): 1. a binary signal detection task with a known lesion location and 2. a more challenging detection-localization task in which the lesion’s specific site among the four predefined candidates was unknown.

##### 3.2.2.1 Binary detection task

Let $f\in R^{N}$ denote the reconstructed image obtained by a specific reconstruction method, where N is the total number of image voxels. In the binary detection task with a known lesion location, the objective is to classify the reconstructed image f as satisfying either a signal-absent hypothesis $H_{0}$ or the signal-present hypothesis $H_{1}$, defined as follows:

$$H_{0}:\;f=f_{b}+n\;(\text{lesion absent}),$$ (7)

$$H_{1}:\;f=f_{b}+f_{s}+n\;(\text{lesion present}),$$ (8)

where $f_{b}\in R^{N}$ denotes the background, $n\in R^{N}$ represents noise, and $f_{s}\in R^{N}$ represents the signal (lesion). It is assumed that the lesion location is known and that both the background and lesion amplitudes vary statistically with known distributions. This corresponds to a signal-known-statistically and background-known-statistically (SKS/BKS) task with the location-known-exactly (LKE) condition. The BKS formulation is adopted over the simpler background-known-exactly assumption to better reflect realistic object variability. In PACT, signal amplitudes depend not only on the intrinsic optical properties of lesions but also on the local optical fluence, which is determined by photon transport through a stochastically varying background. As a result, even when the lesion location and optical properties are known exactly, the lesion amplitude in the initial pressure distribution varies across samples and cannot be treated as exactly known. By adopting an SKS/BKS task with the LKE condition, the effects of lesion location-dependent optical fluence on lesion detectability can be systematically examined under a specific illumination configuration.

In this study, a widely used linear observer, the CHO, is employed as the model observer. It projects f onto a lower-dimensional channel output vector $v\in R^{Q}$ using a channel matrix $T\in R^{Q\times N}$, where Q denotes the number of channels and Q<N, as follows:^59^

v=Tf. (9)

The CHO test statistic is then computed as

$$t(f)=w^{T}v,$$ (10)

where the CHO template $w\in R^{Q}$ is given by:

$$w={\left[\frac{1}{2}\left(K_{0}+K_{1}\right)\right]}^{-1}\Delta \overset{¯}{v},$$ (11)

with $K_{0}$ and $K_{1}$ denoting the covariance matrices of v under hypotheses $H_{0}$ and $H_{1}$, respectively. Here, $\Delta \overset{¯}{v}=E[v|H_{1}]-E[v|H_{0}]$ represents the difference between the conditional means of v under the two hypotheses. The channel matrix T was defined according to the selected channel function. Since the objects in the proposed datasets contain radially symmetric lesions, Laguerre-Gauss (LG) channels were adopted in this study.^58^ This CHO can be regarded as an approximation of the optimal linear observer, the HO, that emphasizes features most relevant to the detection task,^59^^,^^65^ as described in Sec. 2.3. In practice, the CHO is applied to the regions of interest (ROI) centered on the signal, allowing for adaptive assessment that accounts for anatomical variability.^66^

With the computed t(f), the observer performance is quantified using the AUC as the FOM. For each lesion location, a distinct CHO template and the corresponding test statistics are computed, and the resulting AUC values are used to quantitatively compare the lesion detection performance of different reconstruction methods.

##### 3.2.2.2 Detection-localization task

The detection-localization task introduces greater complexity by requiring identification of an unknown lesion location among several predefined candidate sites. The observer must classify an image f as either lesion-absent, denoted as hypothesis $H_{0}$, or lesion-present at one of $n_{L}$ possible locations, denoted as hypothesis $H_{\ell }$, where $\ell \in \{1,\ldots ,n_{L}\}$ with $n_{L}=4$ in this study. The hypotheses are defined as follows:

$$H_{0}:\;f=f_{b}+n\;(\text{lesion absent}),$$ (12)

$$H_{\ell }:\;f=f_{b}+f_{s,\ell }+n\;(\text{lesion present at location }\ell ),$$ (13)

where $f_{s,\ell }\in R^{N}$ represents the signal (lesion) at the ℓ-th candidate location.

A scanning CHO can be adopted for this detection-localization task.^67^ Instead of being applied to a single ROI as in the binary detection task, the scanning CHO is applied to ROIs extracted from the image f at all $n_{L}$ candidate signal locations, computing a location-specific test statistic for each. Specifically, for each candidate location ℓ, a local test statistic is computed as

$$t_{\ell }(f)=w_{\ell }^{T}[v-c_{\ell }],$$ (14)

where $w_{\ell }=K_{\ell }^{-1}\Delta \bar{v_{\ell }}$ is the location-specific CHO template. Here, $\Delta \overset{¯}{v_{\ell }}=E[v_{\ell }|H_{\ell }]-E[v_{\ell }|H_{0}]$ denotes the difference between the conditional means of the channel outputs under hypotheses $H_{\ell }$ and $H_{0}$, $K_{\ell }=\frac{1}{2}(K_{0,\ell }+K_{1,\ell })$ is the average of the covariance matrices of the channel outputs under the two hypotheses, and $c_{\ell }=\frac{1}{2}(E[v_{\ell }|H_{0}]+E[v_{\ell }|H_{\ell }])$ defines the decision origin, all corresponding to location ℓ.^68^ Among all candidate locations, the maximum test statistic is identified as

$$t_{\max}(f)=\underset{\ell }{\max}t_{\ell }(f),\;r=\arg\underset{\ell }{\max}t_{\ell }(f),$$ (15)

where r denotes the estimated lesion location.

To quantify the detection-localization performance of reconstruction methods, the area under the localization ROC (ALROC) curve is used as the FOM. The localization ROC extends the conventional ROC analysis by incorporating both detection and spatial localization accuracy, plotting the probability of correct localization versus the false positive fraction.^69^

## 4 Benchmarking Study

The generated datasets of objects and corresponding simulated measurement data, together with the procedures for computing both traditional and task-based measures of IQ, constitute a comprehensive benchmarking framework. This framework, along with all associated data, is publicly available to facilitate reproducible research in PACT reconstruction methods.^35^^,^^36^ To demonstrate the utility of the proposed framework, an example benchmarking study was conducted to evaluate the capability of DL-based and physics-based reconstruction methods in compensating for acoustic aberrations in PACT. In this study, it was assumed that both the true objects and the corresponding measurement data were available for determining parameters associated with the DL-based and physics-based methods, while only the measurement data were available for reconstruction during performance evaluation.

### 4.1 Dataset Used in Benchmarking Study

Dataset 2, which incorporates breast acoustic heterogeneity and point-like transducers modeling, was employed in this study. It was systematically partitioned into training, validation, in-distribution (ID) testing, and out-of-distribution (OOD) testing subsets. The ID test set maintained the statistical properties of the training distribution, whereas the OOD test set was designed to evaluate model generalization under distributional shifts. The dataset consists of 4860 samples, categorized by breast tissue density types and lesion presence. All measurements were simulated using a ring-array geometry with 900 uniformly distributed transducers. Additive Gaussian noise was introduced, modeled as independently and identically distributed (i.i.d.) with a standard deviation equal to 1% of the maximum photoacoustic signal amplitude across the dataset.^20^

Table 3 summarizes the details of the dataset division used for this study. Data corresponding to type D breasts, which represent extremely dense tissue where lesion identification is particularly challenging in gold-standard mammography, were reserved for the OOD test set. The remaining breast types (A, B, and C) were used for training, validation, and ID testing. Specifically, a total of 4500 samples from breast density types A, B, and C, distributed in a 1:4:4 ratio to reflect clinical prevalence, were allocated such that 70% were employed for training, 10% for validation, and 20% for ID testing. Lesion prevalence was balanced in the training and validation sets but set to 20% in the ID test set to reflect real-world biopsy-positive rates,^70^ resulting in 1980 lesion-present and 2520 unpaired lesion-absent samples. A total of 360 type D breast samples were used for OOD testing, matching the total number of type A samples used across the training, validation, and ID test sets. Both the ID and OOD test sets contained 180 lesion-present samples, with the OOD test set assuming balanced lesion prevalence.

**Table 3:** Dataset splitting for benchmarking study.

Dataset	Size ($N_{\mathrm{samp}}$)	Breast type ($N_{\mathrm{samp}}$)	Prevalence ($N_{\mathrm{lp}}$, $N_{\mathrm{la}}$)
Training	3150	A (350), B (1400), C (1400)	50% (1575, 1575)
Validation	450	A (50), B (200), C (200)	50% (225, 225)
ID testing	900	A (100), B (400), C (400)	20% (180^a^, 720)
OOD testing	360	D (360)	50% (180^a^, 180)

$N_{\mathrm{samp}}$: Number of samples; $N_{\mathrm{lp}}$: Number of lesion-present samples; $N_{\mathrm{la}}$: Number of *unpaired* lesion-absent samples.

a An equal number of *paired* lesion-absent samples, corresponding to $N_{\mathrm{lp}}$ listed in this table, were additionally used for task-based IQ assessments.

### 4.2 Reconstruction Methods

The study compared representative physics-based and DL-based reconstruction methods. To avoid inverse crime,^71^ a condition in which the same computational model is used to simulate the measurement data and included as part of the image reconstruction process, the reconstructions were conducted on a coarser grid (0.25 mm grid size, 680×680  pixels) than that employed for the forward simulations (0.125 mm grid size, 1360×1360  pixels).

Two representative DL-based reconstruction methods were evaluated, which differ in the domain of operation: data-domain and image-domain learning approaches.

• Data-domain learning method (${\mathrm{DL}}_{\text{data}}$): Inspired by the U-Net-based sinogram enhancement method,^49^ this approach employs a data-to-data U-Net^72^ to map noisy measurements simulated under an acoustically heterogeneous medium to corresponding noiseless measurements obtained under an acoustically homogeneous lossless medium assumption.^45^ The output from the U-Net is subsequently used as input to the time reversal (TR) method for image reconstruction under the assumption of a homogeneous medium.
• Image-domain learning method (${\mathrm{DL}}_{\text{image}}$): Inspired by the DeepMB method,^73^ this method employs an image-to-image U-Net architecture to map time-delayed reconstructions obtained from noisy measurements to the corresponding initial pressure distribution. The network is trained in a supervised manner using the true initial pressure as the target. Unlike some variants of DeepMB, this method does not incorporate SOS encoding. The network output serves as the final reconstruction.

Three physics-based methods were used for comparison: TR, penalized least-squares with total variation regularization (PLS-TV), and joint reconstruction (JR) methods.

• TR method:^45^ This method recovers the initial pressure distribution by backpropagating the measurements in reversed time. The reconstruction was implemented using the k-Wave toolbox, which employs a k-space pseudospectral time-domain scheme to solve the system of coupled acoustic equations. A two-region SOS model with distinct values for water and breast tissue is assumed, while acoustic density and attenuation are maintained homogeneous and equal to water throughout the reconstruction domain. The SOS value for breast tissue was optimized by varying it within the reported range of 1.43 to 1.57  mm/μs in 0.005  mm/μs increments.^74^
• PLS-TV method: This method uses the same two-region SOS model as the TR method and is solved using the fast iterative shrinkage-thresholding algorithm (FISTA).^75^ The regularization parameter was selected through parameter sweeping and set to $1\times 10^{-1}$ based on the minimum MSE between the reconstructed and true initial pressure distributions.
• JR method:^9^ This method jointly estimates the initial pressure and a spatially heterogeneous SOS distribution by solving a constrained optimization problem using support, bound, TV-norm constraints. The TV norms of both the initial pressure and SOS estimates were upper-bounded using thresholds set to the 95th percentile of the corresponding TV-norm distributions computed from the training dataset. Additional details are available elsewhere.^9^

### 4.3 Image Quality Evaluation

Following the evaluation strategies described in Sec. 3.2, both traditional and task-based measures of IQ were employed to quantitatively assess the performance of the reconstruction methods. For traditional IQ measures (Sec. 3.2.1), the MSE and SSIM were computed for the reconstructed images (680×680  pixels) obtained using the DL-based, TR, and PLS-TV methods for both the ID and OOD test sets summarized in Table 3. These metrics are reported as quantitative references alongside visual inspection results. The JR method was excluded from the full test set evaluation due to its high computational cost, which arises from the joint estimation of the SOS and initial pressure. Its evaluation was conducted only on the test samples used in the task-based assessment, which form a representative subset of the full test sets employed for the other reconstruction methods (Sec. 4.2). The corresponding MSE and SSIM values for the JR method are reported alongside visual inspection results but are excluded from cross-method statistical comparisons.

Task-based IQ evaluation was performed to assess the diagnostic efficacy of the reconstructed images in terms of binary lesion detection and localization, as summarized in Table 4.

**Table 4:** Overview of the task-based image quality evaluation.

Task	Binary detection	Detection-localization
ROI size	50×50 pixels	
Observer type	CHO separately trained on ID and OOD sets (180 pairs each)	Scanning CHO using templates derived from binary detection tasks
Data for template estimation	45 pairs^a^ per lesion location (one lesion-present +1 lesion-absent; four locations total)	
Data for testing	Same as data for the template estimation	45 group^b^ per lesion location (one lesion-present +3 lesion-absent; four locations total)
FOM	${\mathrm{AUC}}_{\ell }$ for each lesion location ℓ	ALROC

a Each pair consists of one lesion-present sample and one lesion-absent sample for the same lesion location.

b Each group consists of one lesion-present sample for that lesion location and three lesion-absent samples from the other three locations.

#### 4.3.1 Binary detection task

ROIs were extracted from the reconstructed images at each of the four lesion locations, and the CHO analysis described in Sec. 3.2.2 was independently performed for each lesion location to quantify detection performance separately on the ID and OOD test sets. Specifically, for each lesion location, 45 pairs of lesion-present and lesion-absent ROIs centered on the corresponding lesion location were extracted from reconstructed images in both the ID and OOD test sets. The ROI covered a 50×50  pixel area and was represented as a vector $f\in R^{2500}$ under hypotheses $H_{0}$ (lesion-absent) and $H_{1}$ (lesion-present), as defined in Sec. 3.2.2. Example ROI pairs for the four lesion locations, derived from the true initial pressure distributions, are indicated with green boxes in Fig. 5. The CHO was configured with four LG channels (Q=4) and a Gaussian width of 15 pixels. The CHO test statistics t(f) were computed using observer templates w derived from Eqs. (9) to (11). Template estimation followed a resubstitution approach,^65^ in which the same ROI pairs used to compute the test statistics were also used for the template estimation. The detection performance was subsequently evaluated through ROC analysis using the RJafroc R package^76^ to compute AUC based on the test statistics t(f) for each reconstruction method.

#### 4.3.2 Detection-localization task

As in the binary detection task, ROIs were extracted from the reconstructed images at the four predefined candidate sites, ℓ∈{1,2,3,4}. The scanning CHO analysis described in Sec. 3.2.2 was independently performed for each lesion site to quantify localization performance, specifically identifying the unknown lesion site among the four predefined candidate locations. Unlike the ROI pairs used in the binary detection task, 45 ROI groups for this task were organized by lesion location, with each group consisting of one lesion-present ROI and three lesion-absent ROIs corresponding to the remaining candidate sites, all derived from the same breast. An example group is indicated by the blue box in Fig. 5. The LG channels for the scanning CHO were configured identically to those used in the binary detection task. The location-specific templates $w_{\ell }$ in Eq. (14) were estimated using the ROI pairs employed in the binary detection task. The lesion location r was estimated using $w_{\ell }$ as formulated in Eqs. (14) and (15), and the task performance metric ALROC was computed using the RJafroc R package^76^ for each reconstruction method.

**Fig. 5:**
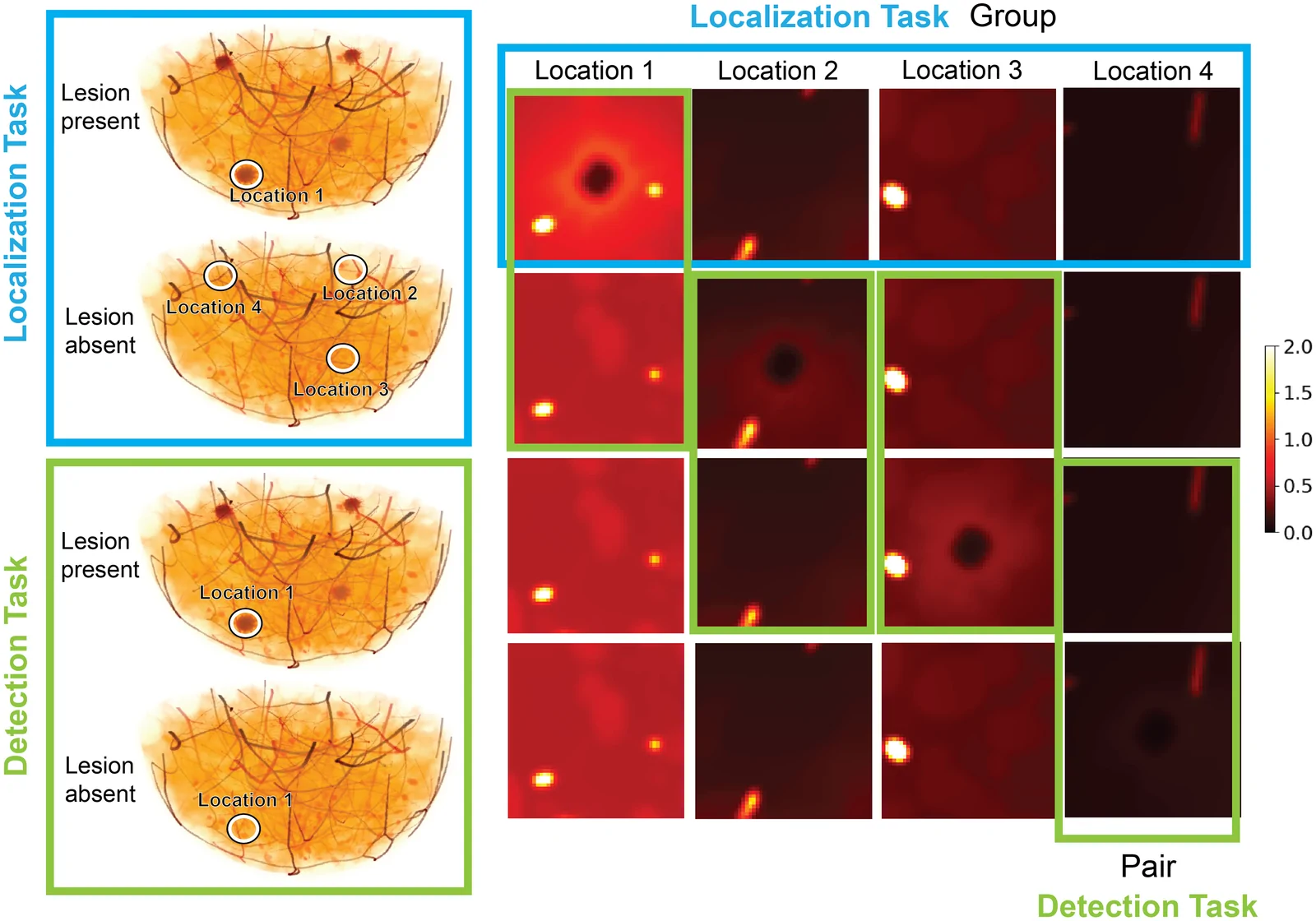
ROI image pairs were used for task-based IQ assessment. The left column shows the ROI extraction locations (circles) on 3D numerical breast phantoms for each task. The right panel displays the corresponding ROI images extracted from the true object. For the localization task, each group (cyan boxes) consists of one lesion-present ROI image at a specific location and three lesion-absent images from the other locations. For the detection task, each pair (green boxes) consists of a lesion-present ROI image and a lesion-absent ROI image from the same anatomical location.

## 5 Benchmarking Study Result

### 5.1 In-Distribution Test Result

Figures 6(a) and 6(b) present representative images reconstructed using the methods considered in this study on the ID test set. Figure 6(a) displays the physical measures of IQ, MSE, and SSIM, and it reveals that the DL-based method (${\mathrm{DL}}_{\text{image}}$) achieved the highest overall IQ, consistent with the visually reduced distortion and sharper anatomical details compared with the other methods. However, as illustrated in the zoomed-in lesion regions in Fig. 6(b), the ${\mathrm{DL}}_{\text{image}}$ method failed to recover clinically significant lesions that were successfully reconstructed by the physics-based methods (TR, PLS-TV, and JR). In particular, although the JR reconstruction retains the lesion best visually, its MSE and SSIM values are not the best among the evaluated methods. This discrepancy highlights the limitation of traditional measures of IQ, which may not fully capture diagnostic utility.

**Fig. 6:**
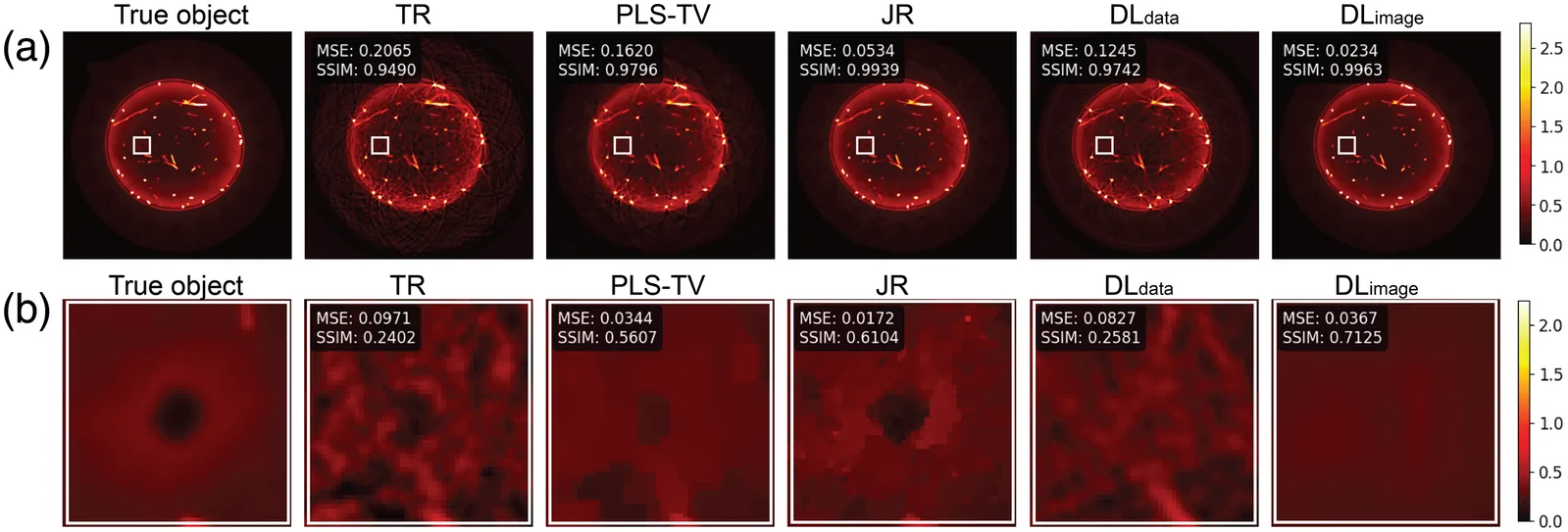
Visual assessment of reconstruction results for a representative sample on the ID test set. The first row (a) presents reconstructed images with annotated MSE and SSIM values, with white boxes marking lesion locations. The second row (b) displays zoomed-in lesion regions. The images, from left to right, show the true initial pressure and reconstructions from TR, PLS-TV, JR, and ${\mathrm{DL}}_{\text{data}}$, ${\mathrm{DL}}_{\text{image}}$. Notably, the JR reconstruction visually recovers the lesion best yet does not attain the best MSE or SSIM scores, whereas ${\mathrm{DL}}_{\text{image}}$ attains favorable MSE and SSIM scores despite failing to recover the lesion. These observations illustrate that commonly used IQ metrics have limited suitability for clinically relevant IQ assessment.

Table 5 reports the MSE and SSIM values for different reconstruction methods on the entire ID test set. Consistent with Fig. 6, the DL-based methods exhibited superior overall IQ.

**Table 5:** Performance evaluation of different reconstruction methods.

Method	MSE	SSIM
TR	0.327±0.098	0.934±0.016
PLS-TV	0.267±0.079	0.975±0.007
${\mathrm{DL}}_{\text{data}}$	0.091±0.040	0.974±0.010
${\mathrm{DL}}_{\text{image}}$	0.017±0.009	0.997±0.002

To comprehensively evaluate diagnostic performance, Figs. 7 and 8 present the results of binary lesion detection analyses on the ID test set. Figure 7 shows the ROC plots for location-specific lesion detection with different reconstruction methods, evaluated using test statistics $t_{\ell }(f)$ derived from the CHOs, while Fig. 8 reports the corresponding AUC values. For comparison, the MSE and SSIM values for the same ID test samples are also included in Fig. 8. The results in Fig. 8 show that traditional metrics (MSE and SSIM) consistently favored the DL-based methods across all lesion locations, exhibiting minimal depth-dependent variation. In contrast, the task-based measure of IQ (AUC) revealed significant depth-dependent performance differences across reconstruction methods. Specifically, at a depth of 10 mm (Locations 1 and 2), AUC values aligned with traditional metrics, indicating superior lesion detectability for DL-based methods. However, at a depth of 20 mm (Locations 3 and 4), MSE and SSIM values did not indicate degraded performance relative to 10 mm, whereas AUC values decreased across all methods, consistent with reduced signal-to-noise ratio due to optical attenuation at greater depths. Notably, the DL-based methods maintained the best MSE and SSIM but showed the lowest AUC at a lesion depth of 20 mm, reflecting their reduced lesion detectability of low-contrast lesions. In contrast, the physics-based methods achieved higher lesion detectability despite inferior MSE and SSIM, with the JR method demonstrating the best task-based performance at both depths.

**Fig. 7:**
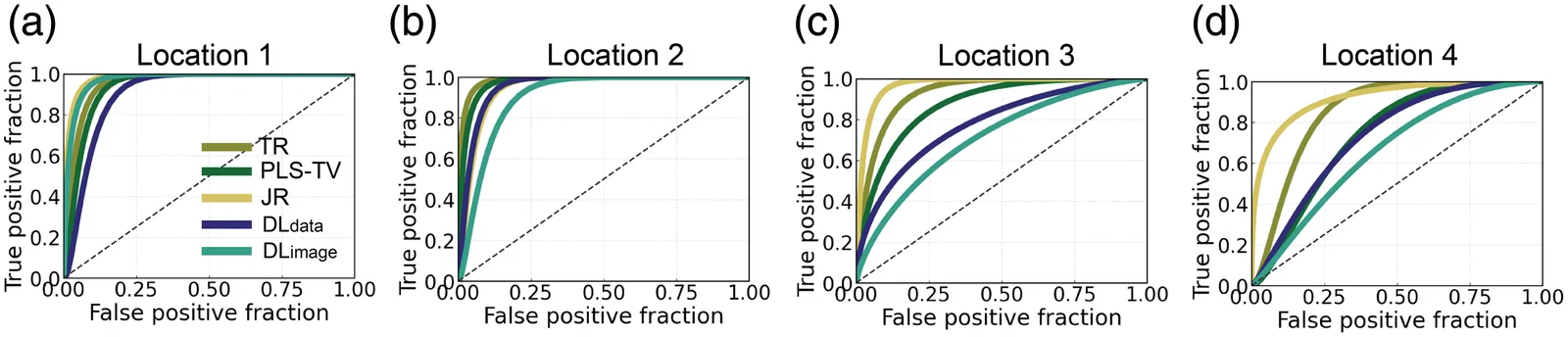
ROC analysis of binary lesion detection performance for reconstruction methods on the ID test set across four lesion locations: Panels (a)–(d) show correspond to locations 1 to 4. The results demonstrate performance degradation from shallow (Locations 1 and 2, 10 mm) to deep (Locations 3 and 4, 20 mm) lesions, where DL-based methods exhibited greater sensitivity to depth-dependent optical attenuation.

Figure 9 presents the results of the lesion localization task, along with the MSE and SSIM values for the ID test set. Consistent with the binary detection results, the DL-based methods yielded the lowest ALROC values despite achieving the highest traditional measures of IQ scores, indicating the poorest localization performance. This deficiency was primarily due to missed lesions at deeper locations, as evidenced in Fig. 8. In contrast, the JR method achieved significantly superior localization performance despite suboptimal MSE and SSIM scores. These findings emphasize the critical importance of evaluating reconstruction methods using both traditional IQ and diagnostic task performance metrics within the proposed framework. It is particularly crucial for DL-based methods that may achieve superior physical measures of IQ while failing to reconstruct clinically significant low-contrast lesions.

**Fig. 8:**
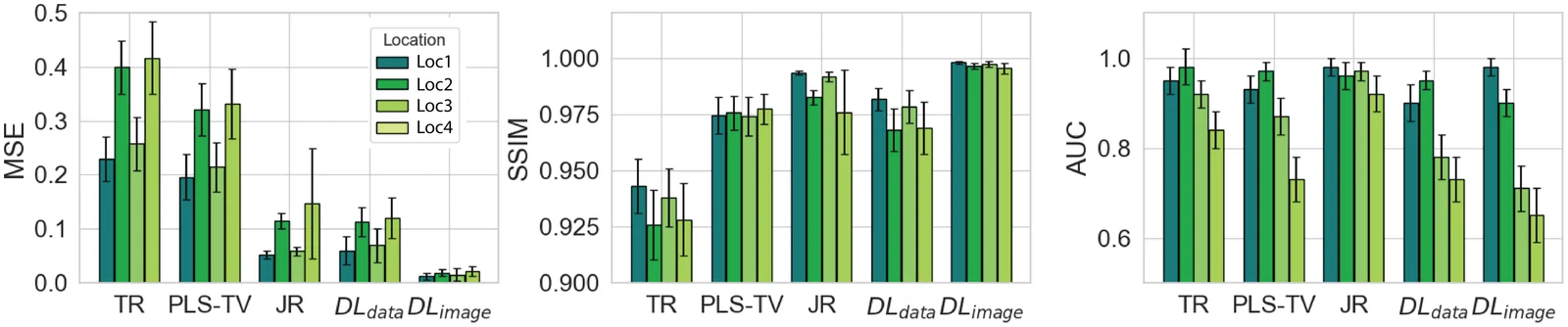
Bar plots showing traditional measures of IQ (MSE and SSIM) and a task-based measure of IQ (AUC) for binary detection tasks at four lesion locations in the ID test set. The results indicate that the traditional metrics failed to capture depth-dependent variations in lesion detectability across methods, whereas AUC clearly revealed performance degradation at greater depths (i.e., Locations 3 and 4), particularly highlighting the reduced lesion detectability of DL-based methods when clinically important low-contrast lesions were missed.

**Fig. 9:**
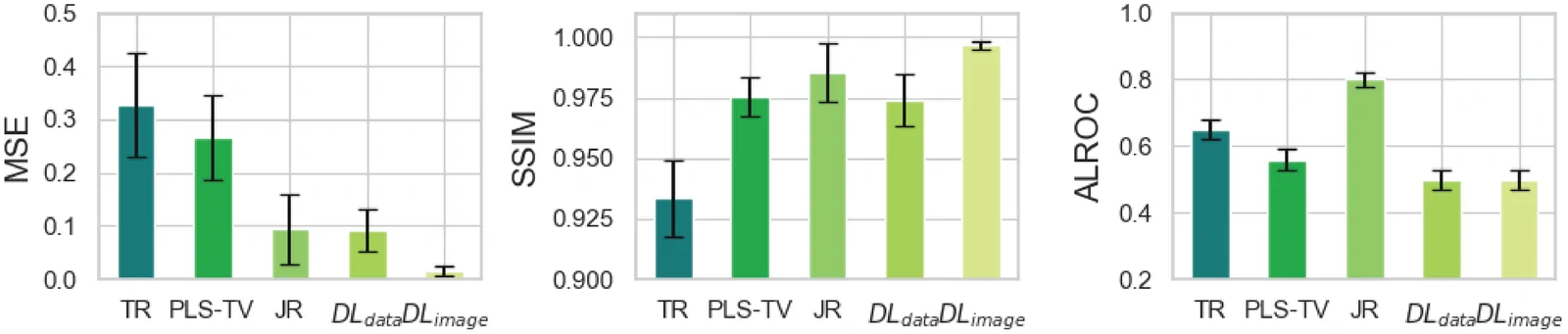
Bar plots of the task-based measures of IQ for lesion localization (ALROC) in the ID test set, with traditional measures of IQ (MSE and SSIM) included for comparison. Results show that the DL-based method achieved the highest traditional metrics values but the lowest ALROC values, indicating the poorest localization performance and highlighting the limitations of traditional metrics in assessing lesion localization.

### 5.2 Out-of-Distribution Test Result

Figure 10 presents bar plots of the MSE and SSIM values for the different reconstruction methods on the OOD test set, with the ID test set included for comparison. The OOD test set consisted exclusively of extremely dense breasts, which are typically considered easier to accurately reconstruct, as evidenced by the improved performance of the physics-based methods from the ID to OOD test results. However, the DL-based methods struggled to generalize to this unseen data distribution, as indicated by degraded traditional measures of IQ metrics. These results suggest that the DL-based approaches exhibited sensitivity to distributional shifts and further highlight the need for meaningful robustness evaluation of DL-based reconstruction methods, particularly regarding their generalizability.

**Fig. 10:**
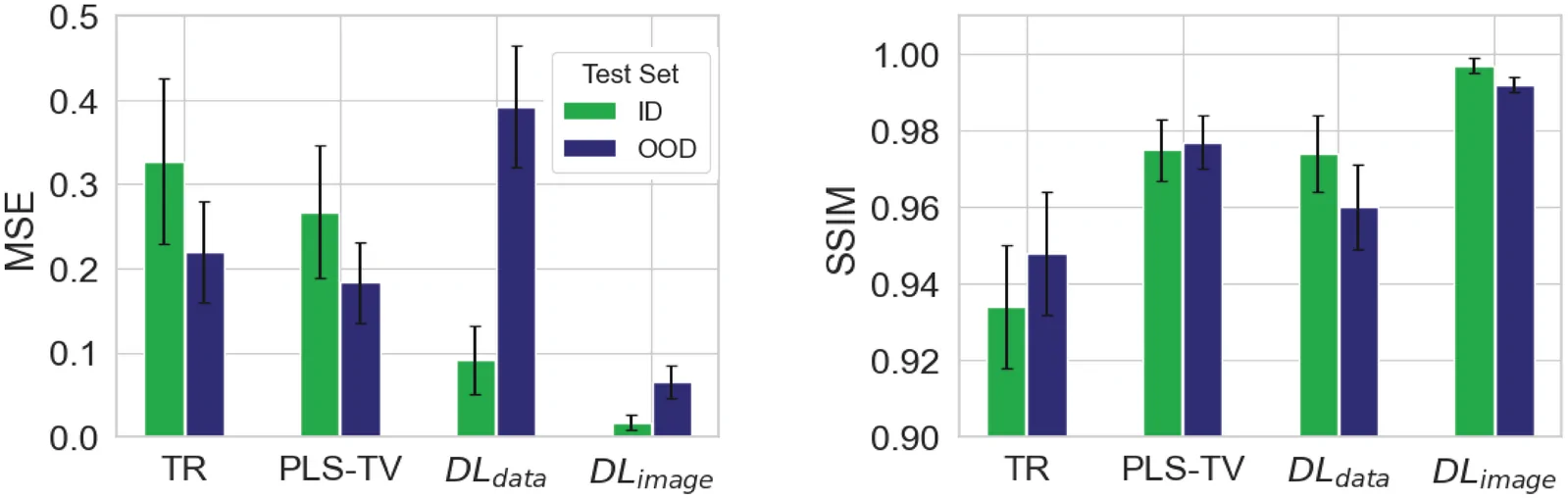
Bar plots comparing MSE (left) and SSIM (right) across reconstruction methods. The quantitative results indicate that the DL-based methods struggled with generalization on the OOD test set, despite dense breasts being generally considered easier to reconstruct, as indicated by the performance trends in the physics-based methods.

Table 6 reports the quantitative results for traditional and task-based measures of IQ on the OOD test set, where bold values indicate the best result per row. In the binary lesion detection task, consistent with the ID test results, traditional metrics suggest that the ${\mathrm{DL}}_{\text{image}}$ method performed well, particularly at Locations 2 and 4. However, its AUC values indicate limited detectability, especially for lesions located at greater depths (Locations 3 and 4, 20 mm). In contrast, the JR method consistently achieved superior location-specific lesion detectability. The results of the lesion localization follow the same trend: the ${\mathrm{DL}}_{\text{image}}$ method yielded the lowest ALROC values, despite the competitive MSE and SSIM scores, indicating that the reconstructed images were less informative for the localization task. Conversely, the physics-based methods, although yielding lower scores on traditional IQ metrics, significantly outperformed ${\mathrm{DL}}_{\text{image}}$ in lesion localization accuracy.

**Table 6:** Quantitative comparison of reconstruction methods (Mean ± std) on the OOD test set.

Metric	Location	TR	PLS-TV	JR	${\mathrm{DL}}_{\text{data}}$	${\mathrm{DL}}_{\text{image}}$
MSE ↓	Location 1	0.159±0.022	0.136±0.023	0.050±0.009	0.354±0.084	0.061±0.016
	Location 2	0.265±0.046	0.219±0.037	0.080±0.014	0.436±0.055	0.064±0.022
	Location 3	0.176±0.026	0.149±0.025	0.055±0.008	0.389±0.074	0.065±0.016
	Location 4	0.273±0.018	0.223±0.017	0.082±0.012	0.390±0.045	0.070±0.022
SSIM ↑	Location 1	0.964±0.007	0.981±0.005	0.996±0.001	0.989±0.002	0.973±0.005
	Location 2	0.940±0.010	0.975±0.006	0.988±0.002	0.952±0.007	0.991±0.002
	Location 3	0.954±0.010	0.978±0.006	0.994±0.001	0.964±0.006	0.993±0.002
	Location 4	0.935±0.013	0.973±0.008	0.986±0.003	0.952±0.010	0.991±0.003
AUC ↑	Location 1	0.97±0.02	0.97±0.02	0.98±0.01	0.96±0.03	0.95±0.02
	Location 2	0.96±0.02	0.93±0.03	0.96±0.02	0.92±0.03	0.85±0.04
	Location 3	0.90±0.04	0.90±0.04	0.90±0.03	0.72±0.06	0.72±0.05
	Location 4	0.75±0.05	0.71±0.05	0.78±0.05	0.67±0.08	0.65±0.06
ALROC ↑	Total	0.67±0.03	0.60±0.03	0.68±0.03	0.56±0.03	0.44±0.03

## 6 Discussion and Conclusion

To facilitate fair comparison and clinical utility assessment of DL-based methods for PACT, this study proposes a benchmarking pipeline comprising realistic synthetic datasets and strategies to rigorously assess the performance of DL-based acoustic inversion methods. Specifically, the datasets feature breast tissue objects derived from 3D stochastic numerical phantoms that capture anatomical and physiological variability with clinically relevant lesions, paired with measurements generated through high-fidelity virtual imaging that closely mimics real-world conditions. The evaluation strategies combine traditional measures of IQ for quantifying overall image fidelity with task-based measures of IQ for assessing lesion detection and localization performance.

The proposed datasets provide a well-characterized benchmark for the systematic evaluation of PACT reconstruction methods. Compared with existing experimental datasets, such as the forearm experimental data provided in OADAT,^24^ the proposed synthetic datasets offer known ground truth and enable large-scale data generation at relatively low cost through computational modeling. Compared with existing synthetic PACT datasets, which have been generated from segmented anatomical maps,^77^ vascular simulations,^78^ their combinations,^23^ or synthetic forearm models such as those in OADAT,^24^ the proposed datasets are constructed from stochastic breast object models that incorporate clinically relevant anatomical, physiological, and pathological variability. This variability provides a more comprehensive representation of breast tissue characteristics and supports robust feature learning by DL-based models, thereby enhancing their representational power and generalizability. In addition, the proposed datasets employ high-fidelity virtual imaging to model the PACT acquisition process and incorporate controllable levels of acoustic modeling complexity, enabling the effects of individual physical factors on reconstruction performance to be investigated while other conditions are held constant. Furthermore, the inclusion of controlled lesion-present and lesion-absent samples enables task-based IQ assessment using numerical observers, allowing reconstruction methods to be evaluated not only in terms of image-domain fidelity but also in terms of their ability to preserve task-relevant information. Collectively, these characteristics make the proposed datasets particularly well-suited for controlled, task-based benchmarking of PACT reconstruction methods.

The benchmarking results revealed critical limitations of traditional measures of IQ for clinical assessment and highlighted the importance of incorporating task-based measures of IQ. While Table 5 showed that the DL-based methods achieved superior MSE and SSIM scores, Fig. 6 revealed their limited sensitivity to subtle yet clinically significant lesions, in contrast to physics-based methods that exhibited the opposite trend. This discrepancy demonstrates that traditional IQ measures based on pixel-level comparisons may fail to fully capture image features that are relevant to diagnostic performance. Task-based measures of IQ also provide quantitative guidance for optimizing DL-based methods, which can inform their eventual clinical translation. For instance, Figs. 7 and 8 illustrate the impact of lesion depth on detectability, revealing that DL methods exhibited particularly poor performance for low-contrast lesions at greater depths. These insights suggest potential improvements, such as incorporating region-of-interest weighting during training or developing task-specific architectures.

An important limitation of the current framework is its restriction to 2D imaging. Although the 2D objects are derived from stochastic 3D breast phantoms using 3D photon transport simulations, extending the framework directly to 3D presents several practical challenges. First, generating a sufficiently large 3D dataset for model training and evaluation would require large ensembles of stochastic 3D breast phantoms together with corresponding measurement data produced using high-fidelity 3D acoustic simulations, imposing substantial computational and storage demands. Second, training and systematically benchmarking 3D DL-based reconstruction methods would be considerably more computationally intensive than in 2D, while many existing DL-based PACT reconstruction methods have been developed and evaluated primarily in 2D. Third, task-based IQ assessment becomes more challenging in 3D because numerical observer models and associated detection or localization tasks must be formulated for volumetric signals and backgrounds, increasing both the dimensionality of the observer problem and its computational burden.

For these reasons, the present study focuses on establishing and evaluating the proposed benchmarking framework in 2D as an initial investigation. Nevertheless, extension to 3D will be important for addressing more realistic clinical imaging scenarios. Future work will therefore focus on developing a fully 3D benchmarking framework, including the generation of volumetric datasets through high-fidelity virtual imaging studies, the development of 3D task-based IQ assessment methods employing volumetric numerical observers, and the systematic evaluation of 3D DL-based PACT reconstruction methods.

Another limitation of this study is that the CHO templates used in the task-based evaluation were estimated using a resubstitution approach, owing to the limited number of samples available for the observer study. Because CHO performance estimated from a finite number of samples is subject to statistical bias and variability, resubstitution may yield optimistically biased estimates of observer performance.^79^ In future studies, more robust estimation strategies, such as cross-validation or the use of independent training and testing datasets, will be considered to mitigate this potential bias and improve the reliability of task-based performance estimates.

Future work will also incorporate additional modeling complexities to further enhance dataset realism and expand the inclusion of physical and task-based measures of IQ for more comprehensive evaluation strategies. The proposed datasets and evaluation strategies are publicly available through^35^^,^^36^ to support benchmarking efforts and facilitate algorithm development within the photoacoustic imaging community, ultimately advancing the clinical translation of PACT technologies.

## Data Availability

The developed synthetic breast datasets and scripts for the evaluation strategies will be made publicly available through open-access repositories^35^^,^^36^ upon acceptance of the paper.
